# Human Papillomavirus Infection in HIV-1 Infected Women in Catalonia (Spain): Implications for Prevention of Cervical Cancer

**DOI:** 10.1371/journal.pone.0047755

**Published:** 2012-10-30

**Authors:** Valeria Stuardo, Cristina Agustí, José Manuel Godinez, Alexandra Montoliu, Aureli Torné, Antoni Tarrats, Carmen Alcalde, Dolores Martín, Eulalia Fernández-Montoli, Cristina Vanrell, Josefa Solé, Yolanda Canet, José Manuel Marqueta, Jadiyettu Mohamed, Isabel Cuenca, Montserrat Lonca, Guillem Sirera, Elena Ferrer, Pere Domingo, Belen Lloveras, Josep María Miro, Silvia De Sanjosé, Jordi Casabona

**Affiliations:** 1 Centre d'Estudis Epidemiològics sobre les Infeccions de Transmissió Sexual i Sida de Catalunya (CEEISCAT), Institut Català d'Oncologia (ICO), Agència Salut Pública de Catalunya (ASPC), Generalitat de Catalunya, Badalona, Spain; 2 Departament de Pediatria, d'Obstetrícia i Ginecologia i de Medicina Preventiva i de Salut Pública, Facultat de Medicina, Universitat Autònoma de Barcelona (UAB), Bellaterra, Cerdanyola del Vallès, Spain; 3 Unit of Infections and Cancer, Catalan Institute of Oncology – ICO-Bellvitge Biomedical Research Institute (IDIBELL), L'Hospitalet de Llobregat, Barcelona, Spain; 4 Hospital Clínico - Institut d'Investigacions Biomèdiques August Pi i Sunyer (IDIBAPS), Barcelona, Spain; 5 Hospital Universitario Germans Trias i Pujol, Badalona, Spain; 6 Hospital Universitario de Bellvitge, Hospitalet de Llobregat, Spain; 7 Hospital de Mataró, Mataró, Spain; 8 Corporació Sanitària Parc Taulí, Sabadell, Spain; 9 Hospital General de L'Hospitalet, Hospitalet de Llobregat, Spain; 10 Hospital de Palamós, Palamós, Spain; 11 Hospital Comarcal del Alt Penedès, Vilafranca del Penedès, Spain; 12 Hospital de la Santa Creu i Sant Pau, Barcelona, Spain; 13 Hospital del Mar, Barcelona, Spain; 14 Centro de Investigación Biomédica en Red (CIBER) Epidemiología y Salud Pública (CIBERESP), Barcelona, Spain; 15 Red Temática de Investigación Coorporativa en Cáncer (RTICC), Barcelona, Spain; 16 Escuela de Salud Pública (ESP), Facultad de Medicina, Universidad de Chile, Santiago, Chile; Vanderbilt University, United States of America

## Abstract

**Background:**

High-risk human Papillomavirus infection is a necessary factor for cervical squamous intraepithelial lesions and invasive cervical cancer. In HIV-1-infected women, HPV infection is more prevalent and a higher risk of cervical cancer has been identified. We aimed to calculate the prevalence of infection by HR-HPV, determine the factors associated with this infection and abnormal cytology findings and to describe the history of cervical cancer screening in HIV-1-infected women.

**Methods:**

We enrolled 479 HIV-1–infected women from the PISCIS cohort. Each patient underwent a gynecological check-up, PAP smear, HPV AND Hybrid capture, HPV genotyping, and colposcopy and biopsy, if necessary. We applied questionnaires to obtain information on sociodemographic, behavioral, clinical, and cervical screening variables. We present a cross-sectional analysis.

**Results:**

Median age was 42 years. The prevalence of HR-HPV infection was 33.2% and that of high-grade squamous intraepithelial lesions (HSIL) was 3.8%. The most common genotypes were 16(23%), 53(20.3%), and 52(16.2%). The factor associated with HR-HPV infection was age <30 years (odds ratio[OR],2.5; 95%confidence interval[CI],1.1–5.6). The factors associated with the presence of HSIL or low-grade squamous intraepithelial lesions (LSIL) were CD4T-lymphocyte count <200cells/mm^3^ versus >500cells/mm^3^ (OR,8.4; 95%CI,3.7–19.2), HIV-1 viral load >10,000copies/mL versus <400copies/mL (OR,2.1; 95%CI,1.0–4.4), and use of oral contraceptives (OR,2.0; 95%CI,1.0–3.9). Sixty percent of HIV-1–infected women had had one Pap smear within the last 2 years.

**Conclusions:**

The high prevalence of HPV infection and cervical lesions in the HIV-1–infected population in Catalonia, as well as the low coverage and frequency of screening in this group, means that better preventive efforts are necessary and should include vaccination against HPV, better accessibility to screening programs, training of health care professionals, and specific health education for HIV-1–infected women.

## Introduction

High-risk human papillomavirus infection (HR-HPV) is a prerequisite for the development of squamous intraepithelial lesions (SIL) and invasive cervical cancer (ICC). Several epidemiological and molecular studies have established a causal association between HPV and ICC [1], [2].

ICC accounts for 9.8% of all human cancers, and every year 500,000 new cases are diagnosed throughout the world. Around 280,000 of these women die, and at least 80% of these deaths occur in developing countries [3]. HPV 16, 18, 31, 33, 35, 45, 52, and 58 cause more than 90.0% of all cases of ICC [4].

Spain has one of the highest prevalences of HIV-1 infection in Western and Central Europe [5]. In 2006 in Catalonia, 32,932 individuals aged 15 to 64 years were infected by HIV-1. The percentage of cases of AIDS diagnosed in women increased from 14.2% in 1986 to 21.9% in 2008. ICC was the defining disease in 4.9% of women diagnosed with AIDS during 1994–2008 [6].

In Spain, the annual incidence of ICC in women of all ages is 6.3 per 100,000 inhabitants and mortality is 1.9 per 100,000 inhabitants. In young women (15 to 44 years), the incidence of ICC is 6.6 per 100,000, making it the second most common cancer after breast cancer [7].

HIV-1–infected women are more susceptible to developing HPV infection and SIL [8], [9]. In Catalonia, the prevalence of HPV infection in HIV-1–positive women exceeds 40%, much higher than the 10% observed in the general population [10]. Similarly, several studies have confirmed that, in Catalonia, the incidence of ICC in HIV-1–infected women is high, with an incidence ratio of 18.5 between HIV-1–infected women and HIV-negative women aged between 20 and 49 years [11], [12].

Several authors have discussed the question of whether the high prevalence of HPV infection and ICC in HIV-1–infected women varies depending on the effectiveness of cervical screening programs [13], [14].

With the aim of studying the association between HIV-1 infection and HR-HPV in our setting, we selected a subcohort of HIV-1–infected women from the PISCIS cohort (Project for Electronic Clinical-Epidemiologic Follow-up of HIV-1 Infection and AIDS). The objectives of the present study were to estimate the prevalence of HR-HPV infection and SIL and associated factors, to describe clinical-epidemiologic characteristics, and examine the history of cervical cancer screening in this population.

## Methods

### Design

The present study was designed as a cohort of HIV positive women from 9 hospitals included in PISCIS cohort. The presented manuscript only shows a cross-sectional analysis of base line data obtained during the first visit of the participating women to the gynecologist. The recruitment period was from September 2007 to March 2009

### Study population

The study population was an opportunistic sample taken from patients included in the PISCIS cohort [15]. The study included a non-discriminatory inclusion, according to degree of immunosuppression, evolution of HIV infection and treatment. All women seeking medical attention at the HIV Unit during the study period were offered to participate. Patients who did not agree to participate were excluded. The participants were referred once or twice per year to the gynecology service of the hospital where they were assisted.

Nine centers from Catalonia were responsible for recruiting and following the women who participated in this study. The hospitals were Hospital Clínico-IDIBAPS (Barcelona), Hospital Universitario de Bellvitge (Hospitalet de Llobregat), Hospital de Mataró (Mataró), Corporació Sanitària Parc Taulí (Sabadell), Hospital Universitario Germans Trias i Pujol (Badalona), Hospital General de L'Hospitalet (Hospitalet de Llobregat), Hospital de Palamós (Palamós), Hospital Comarcal del Alt Penedès (Vilafranca del Penedès), and Hospital de la Santa Creu i Sant Pau (Barcelona).

With an alpha error of 0.05, a hypothetical prevalence of HPV of 45%, and a power of 80% for an odds ratio greater than or equal to 1.9 in the risk factors study, the necessary sample size was calculated to be approximately 500 women. The sample n = 500 considered an oversizing of 10% for attrition (losses) of the sample, the power of 80% was considered for the original sample without oversizing.

The ethics committees of the participating hospitals approved the study and patients gave their signed informed consent to participate. A total of 479 HIV-1–infected patients were recruited during the study period.

### Data collection instruments: questionnaires

Recruitment was carried out by internists who invited the patient to participate in the project and obtained the informed consent, they derived the recruited women to the gynecologist participating in the study and communicated to the coordinating center the number of women who had been recruited for the study.

The study was very well accepted among the women participating in the PISCIS Cohort and only 2 women refused to participate.

At the first visit, a clinical-epidemiologic questionnaire consisting of 40 questions to obtain information on sociodemographic, behavioral, clinical, and cervical screening variables, was administered by the gynecologist. In addition, the questionnaire included 9 questions on screening history; these 9 questions were completed by the gynecologist from medical records.

### Collection and analysis of biological samples

All the patients on the first visit underwent a gynecological check-up and two biological samples were obtained: a first sample was taken for a conventional or liquid cytology test (PAP smear) for subsequent processing at the hospital. A second sample was taken to determine the presence of HPV DNA using a second-generation hybrid capture assay (Digene HC2 DNA test, QIAGEN Inc., Valencia, California, USA) and to genotype the virus (LINEAR ARRAY HPV Genotyping Test, Roche Diagnostics Corporation, Indianapolis, Indiana, USA). The second group of samples was processed at the HPV laboratory of the Cancer and Infections Unit (Catalan Oncologic Institute, Barcelona, Spain). The frequency of the follow-up visits and treatment depended on the results of the diagnostic tests: patients with normal cytology results and negative HPV DNA results had a follow-up visit at 12 months. Patients with an abnormal cytology result or positive HR-HPV, or both underwent colposcopy. Irrespective of the results of colposcopy, patients attended a follow-up visit at 6 months. In cases of an abnormal colposcopy result, a cervical biopsy was performed according to the internal procedures of each hospital. All those patients with cervical lesions were treated according to care protocols.

The HC2 technique identifies HPV DNA using in vitro hybridization with specific RNA probes and can detect 13 types of HR-HPV (16, 18, 31, 33, 35, 39, 45, 51, 52, 56, 58, 59, and 68).

Genotyping by Linear Array amplifies and detects a fragment of HPV DNA. The PGMY09/11 primer set targets a 450-bp region of the HPV L1 gene and can identify 37 types of HPV with a low, high, and probable oncogenic risk (6, 11, 16, 18, 26, 31, 33, 35, 39, 40, 42, 45, 51, 52, 53, 54, 55, 56, 58, 59, 61, 62, 64, 66, 67, 68, 69, 70, 71, 72, 73, 81, 82, 83, 84, IS39, and Cp6108).

### HIV data

In order to cross the HIV and HPV co infection data of women participating in the study, data from HIV infection history (CD4 (cells/mm3), CV (copies/mL), treatment, etc.) of each patient were retrieved from the PISCIS Cohort databases this information was taken from the medical records. The data of CD4 and VL measures included in the analysis was the obtained closer to the date of the first visits to the gynecologist in the study of each patient, the latest CD4 and VL that were in medical records.

### Statistical analysis

Quantitative variables were described using the median (interquartile range [IQR]); qualitative variables were described using percentages. Groups were compared using the Pearson χ^2^ test; when the expected frequencies were less than 5, the Fisher exact test was applied. Univariate and multivariate logistic regression models were applied to identify possible factors associated with HR-HPV infection and the presence of abnormal cytology findings (high-grade squamous intraepithelial lesions [HSIL] and low-grade squamous intraepithelial lesions [LSIL]). The measure of association used was the odds ratio (OR), and a p-value of <0.05 was considered statistically significant. Data were analyzed using SPSS version 17.0.

## Results

Of the 11,374 patients comprising the PISCIS cohort, 2,519 (22.1%) were women. The population of the present study comprised 479 HIV-1–infected women (19% of the total number of women in PISCIS).

Table 1 shows the main sociodemographic, behavioral, clinical, and history of cervical cancer screening variables. The median age of the participants was 42 years. The main route of transmission of HIV-1 was heterosexual relations (75.2%). Of all participating women, 66 (13.8%) were not in treatment and 413 (86.2%) were in treatment. From those in treatment, 383 (92.7%) were on HAART. Highly active antiretroviral therapy (HAART) was defined as a regimen composed of at least 3 antiretroviral drugs. The median CD4 T-lymphocyte count was 480 cells/mm^3^ (IQR, 331–702) and the median viral load was 50 copies/mL (IQR, 40–584). Median time on treatment was 90 months (IQR, 43–132), and the median time from diagnosis of HIV-1 infection documented in the medical records was 119 months (IQR, 59–191)

**Table 1:** Main sociodemographic, behavioral, and clinical characteristics of the study population (n = 479).

*Characteristic* †	*n (%)*
*Age (years)*	
<30	37 (7.7)
30–40	164 (34.3)
>40	278 (58.0)
Total	479 (100)
*Place of birth*	
Spain	348 (72.7)
Other	131 (27.3)
Total	479 (100)
*Transmission group*	
IVDU	97 (21.5)
Heterosexual	340 (75.2)
Other	15 (3.3)
Total	452 (100)
*Age at first sexual intercourse (years)*	
≤18	379 (79.5)
>18	98 (20.5)
Total	477 (100)
*Smoking*	
No	134 (28.0)
Ex-smoker	85 (17.8)
Yes	259 (54.2)
Total	478 (100)
*Number of pregnancies*	
0	68 (14.3)
01-març	301 (63.1)
≥4	108 (22.6)
Total	477 (100)
*Self-reported STIs*	
Yes	109 (23.5)
No	355 (76.5)
Total	464 (100)
*HAART* a	
Yes	383 (92.7)
No	30 (7.3)
Total	413 (100)
*Pap smear* *	
Negative	357 (74.5)
ASC-US	38 (7.9)
LSIL	66 (13.8)
HSIL	18 (3.8)
Total	479 (100)
*Time since last Pap smear (years)* §	
Never	51 (11.0)
<2	276 (60.0)
02-març	60 (13.0)
>3	75 (16.0)
Total	462 (100)
*Frequency of Pap smear (years)*	
Yearly	210 (50.6)
Every 2–3 years	105 (25.3)
Every 4–5 years	39 (9.4)
Every 6–10 years	34 (8.2)
<1 every 10 years	27 (6.5)
Total	415 (100)

† Present the valid percent, the total sample was n = 479 but there were some missings data.

* Performed at the first visit to the study gynecologist.

§ *medical records*.

a HAART was defined as the combination of at least 3 antiretroviral drugs.

Abbreviations: IVDU, intravenous drug user; STI, sexually transmitted infection; HAART, highly active antiretroviral infection; ASC-US, atypical squamous cells of undetermined significance; HSIL, high-grade squamous intraepithelial lesion; LSIL, low-grade squamous intraepithelial lesion.

With regard to history of cervical cancer screening, 11% of the number of women infected by HIV-1 had never had a Pap smear, only 60.0% had had one Pap smear within the last 2 years, and 50.6% reported a frequency of annual screening.

**Figure 1:**
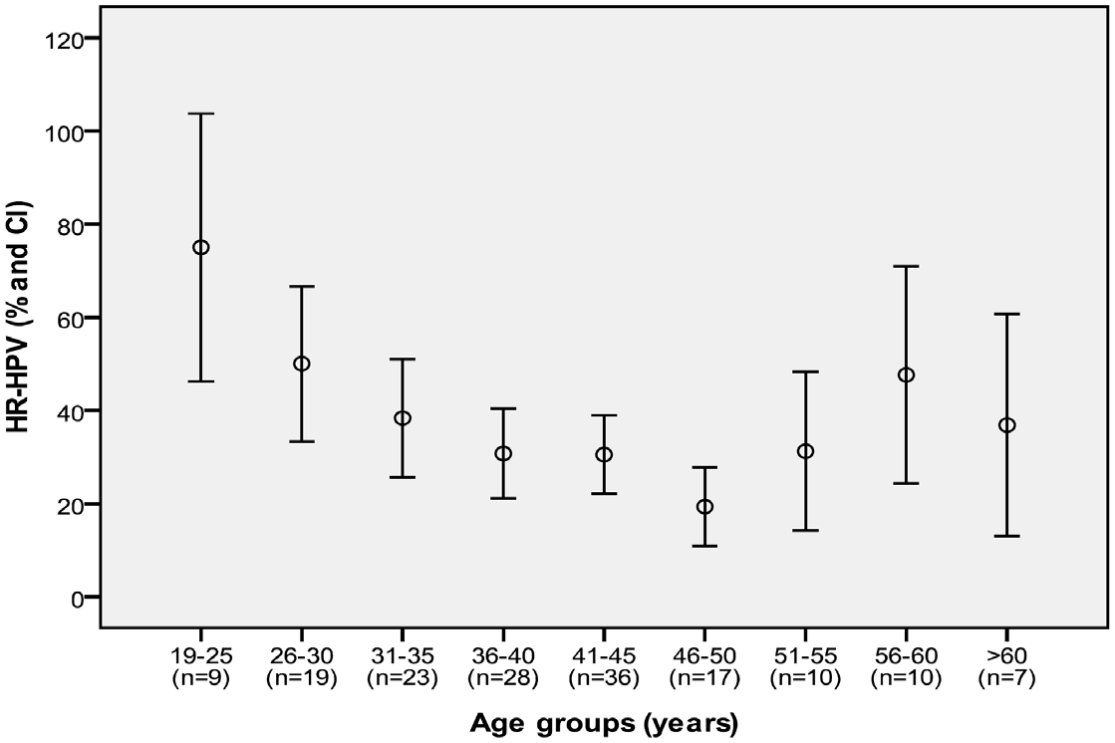
Prevalence curve of HR-HPV infection by age group.

Of the 479 patients comprising the study sample, 159 (33.2%) had a positive result by HC2. The prevalence of infection by HR-HPV according to age group is shown in Figure 1; prevalence was higher among younger women and there was an increase after 50 years.

Genotyping was performed on all patients who were HPV (HR) positive by HC2 (n = 159). Genotyping results were obtained for 148 (93.0%) patients, 11 (7%) were other values 0.

Thirty two (21.6%) women with genotyping results had only one type of HPV, 116 (78.4%) women presented multiple infections. Most of them had between 2 and 4 different types of HPV. (60.1%) The distribution of genotypes is shown in Table 2. HPV16 only appeared as a single genotype causing infection in 5 cases (3,4%). In the remaining cases appeared in combination with other genotypes (Table 2).

**Table 2:** Prevalence single and multiple infections of HPV types in HIV positive women and HPV 16 distribution.

Number of different types*	n (%)§
1	32 (21.6)
2	32 (21.6)
3	37 (25.0)
4	20 (13.5)
5	7 (4.7)
6	9 (6.1)
7	4 (2.7)
8	3 (2.0)
9	1 (0.7)
10	1 (0.7)
11	1 (0.7)
12	1 (0.7)

HPV 16 distribution	n (%)§
16	5 (3.4)
16 y 6	2 (1.3)
16 y 11	2 (1.3)
16 y 59	2 (1.3)
16 y 66	1 (0.7)
16 y 70	1 (0.7)
16,42 y 58	1 (0.7)
16,51 y 53	1 (0.7)
16,11 y 39	1 (0.7)
16,55 y 58	1 (0.7)
16,35 y 84	1 (0.7)
16,62 y 66	1 (0.7)
16,39 y Cp6108	1 (0.7)
16,54 y 61	1 (0.7)
16,59 y 81	1 (0.7)
16,18 y 72	1 (0.7)
16,11 y 42	1 (0.7)
16,31,42 y 53	2 (1.3)
16,18,35 y 62	1 (0.7)
16,6,45,51 y 53	1 (0.7)
16,53,59,67 y Cp6108	1 (0.7)
16,53,55,58,64 y 81	1 (0.7)
16,18,67,68,73 y 84	1 (0.7)
16,18,31,33,51,52 y 54	1 (0.7)
16,31,35,59,72,81 y IS39	1 (0.7)
16,6,40,52,56,61,67,73 y Cp6108	1 (0.7)

* Include LR-HPV, probable and HR-HPV types.

§ % in relation to all women with genotyping (n = 148).

**Figure 2:**
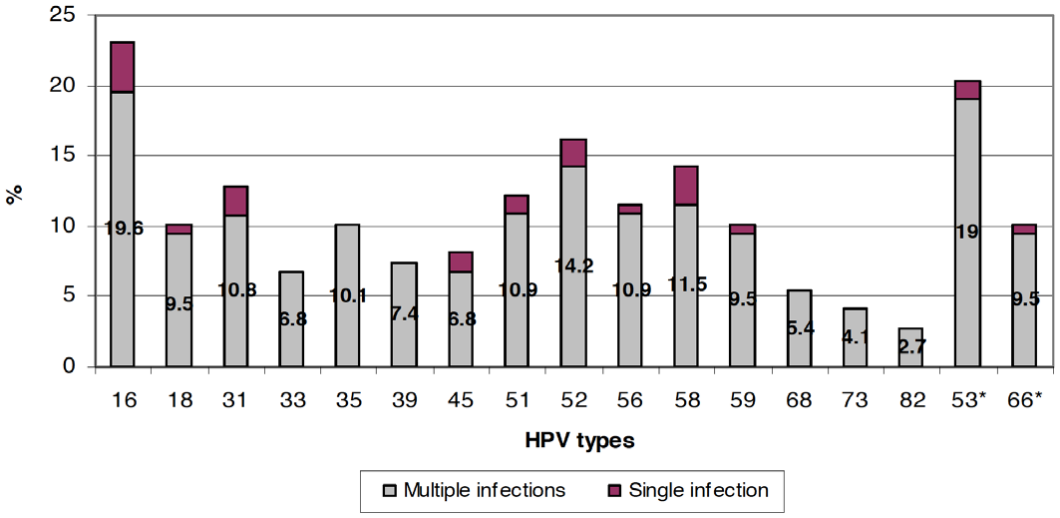
HR-HPV and provable HR-HPV* types-specific prevalence in HIV-1 positive women.

The most prevalent types of HR-HPV and probable HR-HPV, including single and multiple infections, were HPV 16 (23%, n = 34), 53 (20.3%, n = 30), and 52 (16.2%, n = 24) (Figure 2). The most prevalent LR-HPV types were HPV 42 (13.5%), HPV 62 and 84 with 12.8% and 11.5% respectively.

The results of the Pap smear were as follows: atypical squamous cells of undetermined significance (ASC-US), 7.9% (n = 38); LSIL, 13.8% (n = 66); and HSIL, 3.8% (n = 18). Pap smear revealed that 84.8% and 100% of patients with LSIL and HSIL respectively had HR-HPV. Similarly, 34.2% of patients with ASC-US and 20.2% of patients with a normal cytology result had HR-HPV. The most prevalent HR- HPV and probable HR-HPV types in HSIL, including single and multiple infections, were 16 and 53 (37.5% each) and 52 (31.3%), the most prevalent LR-HPV types in HSIL were Cp6108 (37.5%), HPV 84 and 81 (25% each) and HPV IS36 y 67 (18.8% each)

The main differential characteristics in HIV-1–infected patients according to the result of HC2 and cytology are shown in Table 3. Most women with HR-HPV (82.3%) had their first sexual intercourse at age 18 or earlier, and 61.0% of coinfected women had taken oral contraceptives for more than 10 years. As for CD4 T-lymphocyte count, 45.0% of coinfected women had 200–500 cells/mm^3^; most women (64.7%) had a viral load of <400 copies/mL and 48.5% had taken antiretroviral treatment for <60 months.

**Table 3:** Main differential characteristics of the study population according to the results of HC2 and Pap smear.

*Characteristic*	*HPV− (HR)* *n (%)*	*HPV+ (HR)* *n(%)*	*p-value* *	*Pap smear normal* *n (%)*	*Pap smear* ** *abnormal* *n (%)*	*p-value* *
*Age (years)*						
<30	15 (4.7)	22 (13.8)	0.001	25 (6.3)	12 (14.3)	0.05
30–40	107 (33.4)	57 (35.8)		137 (34.7)	27 (32.1)	
>40	198 (61.9)	80 (50.4)		233 (59.0)	45 (53.6)	
*Marital status*						
No stable partner	163 (51.3)	93 (58.9)	0.117	198 (50.4)	58 (69.9)	<0.01
Stable partner	155 (48.7)	65 (41.1)		195 (49.6)	25 (30.1)	
*Age at first sexual intercourse (years)*						
≤18	249 (78.1)	130 (82.3)	0.280	308 (78.2)	71 (85.5)	0.131
>18	70 (21.9)	28 (17.7)		86 (21.8)	12 (14.5)	
*Number of sexual partners to date*						
≤5	174 (54.7)	82 (52.9)	0.821	219 (55.7)	37 (46.2)	0.343
06-oct	49 (15.4)	27 (17.4)		63 (16.0)	13 (16.3)	
nov-20	52 (16.4)	22 (14.2)		57 (14.6)	17 (21.3)	
>20	43 (13.5)	24 (15.5)		54 (13.7)	13 (16.2)	
*Number of sexual partners during the last 6 months*						
None	84 (26.9)	43 (27.9)	0.500	104 (26.9)	23 (29.1)	0.004
1	210 (67.3)	98 (63.6)		262 (67.7)	46 (58.2)	
>1	18 (5.8)	13 (8.5)		21 (5.4)	10 (12.7)	
*Use of condom (stable partner)* §						
Always	117 (57.6)	58 (61.1)	0.460	148 (58.7)	27 (58.7)	0.547
Sometimes	49 (24.2)	17 (17.9)		58 (23.0)	8 (17.4)	
Never	37 (18.2)	20 (21.0)		46 (18.3)	11 (23.9)	
*Use of condom (casual partner)* §						
Always	26 (65.0)	13 (61.9)	0.820	28 (60.8)	11 (73.3)	0.491
Sometimes	7 (17.5)	3 (14.3)		9 (19.6)	1 (6.7)	
Never	7 (17.5)	5 (23.8)		9 (19.6)	3 (20.0)	
*Time since oral contraceptives were last taken (years)*						
<1	8 (3.8)	10 (10.0)	0.005	12 (4.9)	6 (9.8)	0.238
01-maig	15 (7.2)	17 (17.0)		23 (9.3)	9 (14.8)	
06-oct	32 (15.4)	12 (12.0)		37 (15.0)	7 (11.5)	
>10	153 (73.6)	61 (61.0)		175 (70.8)	39 (63.9)	
*CD4 T lymphocytes (cells/mm^3^)*						
<200	16 (5.3)	27 (17.9)	<0.01	23 (6.1)	20 (25.6)	<0.01
200–500	128 (42.1)	68 (45.0)		160 (42.4)	36 (46.2)	
>500	160 (52.6)	56 (37.1)		194 (51.5)	22 (28.2)	
*HIV-1 viral load (copies/mL)*						
<400	225 (79.2)	88 (64.7)	0.002	266 (76.7)	47 (64.3)	0.006
400–5,000	28 (9.9)	17 (12.5)		37 (10.7)	8 (11.0)	
5,000–10,000	9 (3.2)	4 (2.9)		12 (3.5)	1 (1.4)	
>10,000	22 (7.7)	27 (19.9)		32 (9.1)	17 (23.3)	
*Time on treatment (months)*						
<60	74 (26.5)	65 (48.5)	<0.01	100 (29.5)	39 (52.7)	<0.01
60–120	107 (38.4)	32 (23.9)		129 (38.1)	10 (13.5)	
>120	98 (35.1)	37 (27.6)		110 (32.4)	25 (33.8)	

* X^2^ test for linear trend.

** (low-grade squamous intraepithelial lesion and high grade squamous intraepithelial lesion).

§ during the last 6 months.

The only factor associated with HR-HPV infection was age: women under 30 years had an OR of having HR-HPV infection that was 2.5 (95% CI, 1.1–5.6) times greater than that of women aged >40 (Table 4). The factors associated with a cytology finding of LSIL or HSIL were CD4 T-lymphocyte count <200 cells/mm^3^ versus >500 cells/mm^3^ (OR, 8.4; 95% CI, 3.7–19.2), viral load >10,000 copies/mL versus <400 copies/mL (OR, 2.1; 95% CI, 1.0–4.4), and use of oral contraceptives (OR, 2.0; 95% CI, 1.0–3.9) (Table 5).

**Table 4:** Multivariate analysis† of the characteristics associated with high-risk HPV infection.

*Characteristic*	*OR (95% CI)*	*p-value*
*Age (years)*		
<30	2.5 (1.1–5.6)	0.026
30–40	1.2 (0.8–2.0)	0.293
>40	1	-
*Age at first sexual intercourse (years)* *		
≤18	1.2 (0.7–2.2)	0.376
>18	1	-
*Number of sexual partners to date* *		
≤5	1	-
06-oct	1.3 (0.7–2.5)	0.286
nov-20	1.0 (0.5–2.0)	0.833
>20	1.2 (0.6–2.4)	0.573
*Marital status* *		
No stable partner	1	-
Stable partner	0.8 (0.5–1.4)	0.600
*Use of condom* *		
Yes	1	-
No	1.0 (0.4–2.4)	0.858

† The model included those variables that were statistically significant in the univariate analysis (p-value<0.05), those with a p-value lower than 0.10, and those that were considered clinicoepidemiologically important.

* Adjustment variables introduced in the final model.

**Table 5:** Multivariate analysis† of the characteristics associated with an abnormal cytology (low-grade and high-grade squamous intraepithelial lesion).

*Characteristic*	*OR (95% CI)*	*p-value*
*CD4 count (cells/mm^3^)*		
<200	8.4 (3.7–19.2)	<0.01
200–500	1.7 (0.9–3.3)	0.061
>500	1	-
*HIV-1 viral load (copies/mL)*		
<400	1	-
400–5,000	1.5 (0.6–3.5)	0.350
5,000–10,000	0.4 (0.04–3.5)	0.417
>10,000	2.1 (1.0–4.4)	0.045
*Smoker* *		
No	1	-
Yes	1.5 (0.7–3.0)	0.218
*Oral contraceptives* *		
Yes	2.0 (1.0–3.9)	0.030
No	1	-

† The model included those variables that were statistically significant in the univariate analysis (p-value<0.05), those with a p-value lower than 0.10, and those that were considered clinicoepidemiologically important.

* Adjustment variables introduced in the final model. Not include ex-smokers.

## Discussion

Our study is the first multicenter study performed in Catalonia to provide data on the prevalence of HR-HPV infection, cervical lesions, and the distribution of high-risk genotypes present in the HIV-1–infected population. We also report data on history of cervical cancer screening.

In HIV-1–infected women, viral persistence is greater and clearance of HPV infection is lower than in immunocompetent women; therefore, the prevalence of HPV infection observed in this population is greater than in the general population, and the risk of progression to high-grade lesions and cancer is also greater [16], [17], [18]. We found prevalence of HR-HPV infection of 33.2%. Although this prevalence is much higher than that observed in general population in Spain (>10%) [19] but is lower than that found in other Spanish studies involving HIV-1–infected women, which show a prevalence >40% [20], [10]. This discrepancy with other studies in HIV women performed in Spain could be explained because the prevalence of HPV varies with age and the median age in our population was higher (42 years) and/or differences in the detection technique.

In the present study, 78.4% of HIV-1–infected women had multiple infections. Irrespective of the technique used to detect HPV DNA, these data are consistent with the results of several studies showing that HIV-1–infected women not only have a greater prevalence of HR-HPV infection, but that they are also infected by a wider variety of types HPV than immunocompetent women [9], [21].

As HIV-1 and HPV are both transmitted through sexual relations and share risk factors for acquisition associated with sexual practices, it is difficult to address the infections separately. The diversity of genotypes and the high prevalence of multiple infections in HIV-1–infected women can be explained more by the effects of HIV-1–induced immunosuppression (modulation of the immune response to HPV, local immunity, and genetic instability) [22] than by risky sexual behavior [23], [24].

Consistent with the results of previous studies in Spain [9], [10], the most prevalent genotypes found in the present study were 16 (23%), 53 (20.3%), and 52 (16.2%).

Infection by HR-HPV is necessary for the development of SIL and ICC [1]. The prevalence of SIL is consistently higher in HIV-1–infected women than in HIV-1–negative women [9], [16].

When we analyzed the distribution of HPV in HSIL, we found a high prevalence of multiple infections, together with the presence of HPV genotypes with lower oncogenic potential. This is probably because in HIV-1–infected women, whose immune systems are weakened, these types of HPV are more likely to evade the immune system and persist in preneoplasic lesions [16], [17], [18].

According Darwich L et al. [25] there is a high prevalence of HPV 16 and 18 in cervical cancers in HIV positive and negative women in Barcelona. In ICC the HPV 18 was significantly more prevalent in HIV-positive women as opposed to HIV-negative women (14% vs 1%). Our study shows a high prevalence of HPV 16 and 53 in HSIL (37.5% each) in HIV positive women, HPV 18 has a prevalence of 12.5%.

Unlike the general population, HIV positive women have a wide range of genotypes in both HSIL and LSIL, probably by the characteristics of sexual behavior and the reactivation of latent infections, which can encourage infection by different HPV types. Taken into account the high prevalence observed of HPV 16 and 18, it is important to consider anti-HPV vaccination as a primary preventive measure in HIV positive women. Studies evaluating the safety and immunogenicity of the quadrivalent human papillomavirus vaccine in HIV-infected women are in progress and are certainly needed.

Differences in the capacity to evade the immune system have been observed among different HPV types. It has been shown that the infection by HPV 16 would not depend as much on immune status, whereas infection by other less prevalent types would, as these could be enhanced by suppression of the quality of the immune response [26]. The presence of multiple types of HPV in HIV-1–infected women in our study could be associated with a greater risk of cervical lesions, although we would need to evaluate follow-up data and thus establish the contribution of genotypes with a low oncogenic potential to the development of HSIL and ICC in this group.

Data from the questionnaires and the clinical records show that 75.2% of women participating in the present study became infected by HIV-1 as a result of heterosexual relations. The high level of promiscuity in the study population—most participating women had their first sexual intercourse before age 18 and the proportion of monogamous relationships was low—could explain why the most common variables associated both with HR-HPV infection and with the presence of abnormal cytology findings were not significant in this study.

The prevalence of HPV infection decreases with age [27]. Younger women present a higher prevalence of infection, due to the characteristics of their cervical epithelium and sexual behavior. We observed that the pattern of infection by age in HIV-1–infected women seems to follow a curve very similar to that observed in the general population, although with a higher intensity, namely, younger women had a higher prevalence and risk of infection by HR-HPV (Figure 1). We also observed an increase in the prevalence of infection in the upper age range; this observation has also been made in other populations and may be due to several factors, such as changes in sexual behavior in middle-aged women, high rates of persistence of HPV, or a weakened immune system [28], [29].

The relationship between antiretroviral therapy and the natural history of HPV infection remains controversial. Whereas some studies have not found a clear association between antiretroviral therapy and the reduction in the prevalence of cervical lesions and HPV infection [8], [30], others point to the contrary in women who receive antiretroviral therapy [31], [32]. In the present study, we found a clear association between immune status of HIV-1–infected women and detection of HR-HPV and abnormal cytology findings: 25.6% of women with an abnormal cytology finding had a CD4 T-lymphocyte count <200 cells/mm^3^ compared with 6.1% of women with a normal cytology result. A similar association was found for detection of HR-HPV and shorter time of treatment. However, these differences only persisted in the multivariate analysis for abnormal cytology findings but not for detection of HR-HPV—due to the existence of confounders such as age or sexual behavior, also these women might have a poorer follow up in general—and significance disappeared in the multivariate analysis. In any case, longitudinal studies would be necessary to ascertain whether immune reconstitution induced by antiretroviral therapy has an effect on the development and progression of cervical lesions in this group, as well as on the incidence and persistence of HR-HPV infection.

The Catalonian Cervical Cancer Screening Protocol recommends that HIV-1–infected women undergo 2 Pap smear separated by an interval of 6 months or 1 Pap smear with colposcopy. In addition, women should have an annual Pap smear, or more frequent Pap smears if their CD4 T-lymphocyte count is <500 cells/mm^3^ or cytology findings are abnormal. The present study revealed inadequate coverage and frequency of cervical cancer screening in HIV-1–infected women: 60.0% had a Pap smear within the last 2 years and 50.6% reported a frequency of annual screening. The data showed an inadequate screening in HIV positive women. It is necessary to design and to implement specific protocols addressed to vulnerable populations.

Taking into account an estimated incidence of HSIL in the Spanish general population of 10 per 100,000 inhabitants, we would expect to find 0.047 cases of HSIL in 479 women, thus confirming the finding that 18 cases of HSIL in our study sample (n = 479) is much higher than expected in women from the general population who receive regular gynecological care. These findings are consistent with the high incidence of ICC found in HIV-1–infected women in Catalonia [11], [12].

Our study has some limitations. The prevalence of HR-HPV infection was determined based on the sensitivity level set in HC2 for detection of high-risk types, and this limit has been clinically validated in several randomized clinical trials. Higher sensitivity limits or broader-spectrum primers could have revealed more cases of HPV infection. However, these limitations did not affect the objectives of the study: the wide scientific evidence available shows that HR-HPV are the most important types of HPV at an epidemiological level, since they cause more than 99% of cases of ICC in both the general population and the HIV-1–infected population. It has to be taken into account that the cohort included a non-discriminatory sample, according to degree of immunosuppression, evolution of HIV infection and treatment, as a consequence, the population of women with recent infection with HIV was not represented in our study. The sample included only women who had a prolonged history of infection (median, 119 months). In addition, many were taking HAART (92.7%) and the median age was high (42 years). These findings could represent an underestimation of HR-HPV infection and of cervical lesions in this population. On the other hand, the sample was not representative of the total HIV infected population in Catalonia, however, when we contrast our data with the AIDS registry of Catalonia, the clinical epidemiological characteristics did not differ, moreover, the sample came from 9 hospitals that account for 80% of HIV patients in Catalonia.

Finally, we retrieved the data closer to the date of 1st visit to the gynecologist for each patient (at inclusion of patients in the study), which means, the latest CD4 and VL who were in medical records. The time frame was not defined, however, over 80% of records corresponding to the last six months, both CD4 and VL.

In conclusion, we confirmed the high prevalence of HR-HPV infection and cervical lesions in HIV-1–positive patients. HPV16 was the most prevalent type. The most important determinant of HR-HPV infection was age less than 30 years. More abnormal cytology findings were recorded in women with a poorer immune status. Coverage and frequency of cervical cancer screening in HIV-1–infected women in Catalonia is suboptimal, especially considering the high-risk nature of this population. Optimizing prevention of ICC is a priority in these patients.
